# Estimated glomerular filtration rate as an independent predictor of atherosclerotic vascular disease in older women

**DOI:** 10.1186/1471-2369-13-58

**Published:** 2012-07-16

**Authors:** Joshua R Lewis, Wai Lim, Satvinder S Dhaliwal, Kun Zhu, Ee Mun Lim, Peter L Thompson, Richard L Prince

**Affiliations:** 1 University of Western Australia, School of Medicine and Pharmacology, Sir Charles Gairdner Hospital Unit, Perth, Australia; 2 Department of Endocrinology and Diabetes, Sir Charles Gairdner Hospital, Perth, Australia; 3 Department of Renal Medicine, Sir Charles Gairdner Hospital, Perth, Australia; 4 School of Public Health, Curtin University, Perth, Australia; 5 Dept. of Cardiovascular Medicine, Sir Charles Gairdner Hospital, Perth, Australia

## Abstract

**Background:**

Estimated glomerular filtration rate (eGFR) levels have been shown to predict atherosclerotic vascular disease hospitalization and mortality. We sought to investigate the role of renal function in the prediction of 10-year atherosclerotic vascular hospitalization and deaths in an unselected population of elderly women in and compared these predictions to Framingham equations.

**Methods:**

Complete 10-year verified mortality and hospitalization discharge records for atherosclerotic vascular disease was collected for a prospective study of 1,239 unselected female subject’s ≥ 70 from the Calcium Intake Fracture Outcome Study (CAIFOS) with 10 years of follow-up. eGFR was compared to the current Framingham risk scores.

**Results:**

The eGFR at baseline using the Modification of Diet in Renal Disease Study (MDRD) equation was 65.2 ± 14.5 mL/min/1.73 m^2^ and 66.3 ± 13.5 mL/min/1.73 m^2^ using the Chronic Kidney Disease EPIdemiology (CKD-EPI) equation. Over 10 years 30% of participants sustained an ASVD hospitalization or death. For every standard deviation (SD) reduction in eGFR using MDRD the odds ratio (OR) for ASVD hospitalization and deaths increased by 1.34 (1.18-1.53), P < 0.001and 1.31 (1.14-1.50), P < 0.001 in a model adjusted for Framingham 10-year general cardiovascular risk. Addition of eGFR by the MDRD equation to Framingham risk factors improved the net reclassification index by 5.9%, P = 0.018 and the integrated discrimination improvement by 0.010 ± 0.003, P < 0.001 Similar results were seen using the CKD-EPI equation.

**Conclusion:**

Estimated glomerular filtration rate predicts ASVD outcomes independently of Framingham risk score predictions in elderly women and improves clinical prediction particularly of early ASVD.

## Background

Atherosclerotic vascular disease (ASVD) remains a leading cause of morbidity and mortality. The development of equations to estimate renal function using age and serum creatinine has shown that estimated glomerular filtration rate (eGFR) is an important predictor of clinical ASVD outcomes, especially mortality, when eGFR falls below 60 ml/min/1.73 m² [1-3] and is associated with all cause and cardiovascular mortality in general population cohorts [4]. Many studies have been restricted to study of patients with eGFR below 60 ml/min/1.73 m² compared to above 60 ml/min/1.73 m² [5-9]. However the effects of earlier stages of renal function deterioration on ASVD outcomes have been less well studied particularly in the elderly [10]. Thus there is uncertainty as to whether mild renal dysfunction may have adverse cardiovascular effects independent of known risk factors in this population.

Two methods of calculating eGFR have been developed the “175” Modification of Diet in Renal Disease (MDRD) equation [11] and the CKD-EPI equation [2]. In middle aged populations comparison of the MDRD equation to the CKD-EPI equation to predict mortality concluded that the CKD-EPI equation showed some advantages over the MDRD equation [2,12,13].

The study design employed used both the revised MDRD and the CKD-EPI equations to examine the relationship of eGFR to atherosclerotic vascular disease in unselected elderly women using complete adjudicated hospital record discharge data from the Western Australian Data Linkage System. In addition the eGFR calculations were compared to the Framingham general cardiovascular risk model.

## Methods

### Study population

The participants were recruited in 1998 and followed for 10 years. At baseline they entered a 5-year prospective, randomized, controlled trial of oral calcium supplements to prevent osteoporotic fractures [14]. The participants were ambulant and did not have any medical conditions likely to influence 5-year survival. They were only excluded if they were receiving bone-active agent, including hormone replacement therapy. Participants were similar in terms of baseline disease burden and medications compared to the whole population of this age but they were more likely to be from higher socio-economic groups [14]. They were subsequently asked to enter a 5-year follow-up study of ageing.

They were recruited from the Western Australian general population of women aged over 70 years by mail using the electoral roll a requirement of citizenship. Over 99% of Australians of this age are registered on the roll. Of the 5,586 women who responded to a letter inviting participation 1,510 women were willing and eligible and of these 1,500 women were recruited for the study. In the first 5 years of the study participants received 1.2 g of elemental calcium as calcium carbonate daily or a matched placebo. The Human Ethics Committee of the University of Western Australia approved the study.

### Renal function assessment

Baseline renal function was determined in 1,239 women. Serum was collected after an overnight fast and serum creatinine analysed in 2005 using an isotope dilution mass spectrometry (IDMS) traceable Jaffe kinetic assay for creatinine on a Hitachi 917 analyzer (Roche Diagnostics GmbH, Mannheim Germany). The estimated GFR calculated in mL/min/1.73 m^2^ using the revised “175” MDRD study equation was; 175 (standardized serum creatinine (Scr) in mg/dL) ^-1.154^ x (Age) ^-0.203^ x 0.742 with creatinine values entered in mg/dL into the equation [11,15,16]. The estimated GFR calculated using the CKD-EPI equations was; Scr ≤ 0.7 mg/dL = 144 × (Scr/0.7)^-0.329^ × (0.993) ^Age^ or Scr > 0.7 mg/dL = 144 × (Scr/0.7)^-1.209^ × (0.993) ^Age^ with creatinine values entered in mg/dL into the equations [2]. Estimated GFR was separated into Chronic Kidney Disease (CKD) categories as defined by the kidney disease outcomes quality initiative (K/DOQI) classification [17].

### Framingham risk score

The 10-year Framingham general cardiovascular disease risk score was calculated using age, previous diabetes, body mass index, current smoking status and the untreated systolic blood pressure using the equation and estimated regression coefficients developed by D'Agostino et al. 2008 [18].

The Framingham risk score (FRS) equation was;

FRS = 1–0.94833^exp(2.72107*ln(Age)+0.51125*ln(BMI)+2.81291*ln(SBP)+0.61868*(Currentsmoker)+0.77763*(Diabetes)-26.0145)^*.*

The risk scores were then confirmed using the online calculator prepared by R.B. D’Agostino and M.J. Pencina based on the publication by D’Agostino et al. [18].

### Baseline ASVD risk assessment

Previous atherosclerotic vascular disease was determined from the complete hospital discharge data from 1980–1998 and were defined using diagnosis codes from the International Classification of Diseases, Injuries and Causes of Death Clinical Modification (ICD-9-CM) [19]. These codes included: ischemic heart disease (ICD-9-CM codes 410–414); heart failure (ICD-9-CM code 428); cerebrovascular disease excluding hemorrhage (ICD-9-CM codes 433–438); and peripheral arterial disease (ICD-9-CM codes 440–444). The participants provided their previous medical history and current medications verified by their General Practitioner. These data were coded using the International Classification of Primary Care – Plus (ICPC-Plus) method [20]. The coding methodology allows aggregation of different terms for similar pathologic entities as defined by the ICD-10 coding system. These data were then used to determine the presence of diabetes at baseline.

At baseline weight was assessed using digital scales with participants wearing light clothes and no shoes, height was assessed using a stadiometer and the body mass index was calculated in kg/m^2^. Blood pressure was measured in 1,205 participants on the right arm with a mercury column Manometer using an adult cuff after the patient had been seated and resting for at least 5 minutes, the average of 3 such measurements was obtained.

### Incident ASVD outcome assessment

The primary outcome was an atherosclerotic vascular disease event causing hospitalization or death. First-time atherosclerotic hospitalizations were retrieved from the Western Australian Data Linkage System (WADLS) for each of the study participants from 1998 until 10 years after their baseline visit. WADLS provides a complete validated record of every participant’s primary diagnosis at hospital discharge using coded data form all hospitals in Western Australia. Cause of death was retrieved from the coded death certificate using information in Parts 1 and 2 of the death certificate, all diagnosis text fields from the death certificate were used to ascertain the cause(s) of deaths where these data were not yet available from the WADLS. Atherosclerotic events were defined using primary diagnosis codes from the International Classification of Diseases, Injuries and Causes of Death Clinical Modification (ICD-9-CM) [19] and the International Statistical Classification of Diseases and Related Health Problems, 10^th^ Revision, Australian Modification (ICD-10- AM) [21]. These codes included: ischemic heart disease (ICD-9-CM codes 410–414 and ICD-10-AM codes I20-I25); heart failure (ICD-9-CM code 428 and ICD-10-AM code I50); cerebrovascular disease excluding hemorrhage (ICD-9-CM codes 433–438 and ICD-10-AM codes I63-69, G45.9); and peripheral arterial disease (ICD-9-CM codes 440–444 and ICD-10-AM codes I70-74).

### Statistical analysis

The primary outcome was the odds ratio of the time to first atherosclerotic vascular hospitalization or death up to ≤ 10 years after the baseline visit. Covariates were entered into the model as continuous variables with the exception of current smoking and diabetes, which were entered as dichotomous (y/n) variables. All continuous variables were naturally logarithmically transformed to improve discrimination and calibration of the models and to minimize the influence of extreme observations. No interactions between baseline confounders were detected. The effect of eGFR on reclassification of risk was assessed using net reclassification improvement and integrated discrimination improvement [22,23]. Using the Framingham risk factors participants were classified into three 10-year risk categories of ASVD, low (< 15%), intermediate (15%-30%) or high (≥ 30%) for ASVD hospitalizations and deaths. These risk categories are based on the median predicted risk by Framingham risk factors (30%). The participants were then reclassified into new risk categories with the addition of eGFR to the model and the net reclassification improvement (NRI) and integrated discrimination improvement (IDI) were calculated. Results are presented as odds ratio (OR) and associated 95% confidence intervals. P values less than 0.05 in two tailed testing were considered statistically significant. The data was analysed using SPSS (version 15; SPSS Inc., Chicago, IL), STATA (version 11 StataCorp LP, College Station, TX) and SAS (Version 9, SAS Institute Inc., Chicago, IL).

## Results

The mean age of the 1,239 participants was 75.2 ± 2.7 years and the mean eGFR by the MDRD and the CKD-EPI equations was 65.2 ± 14.5 and 66.3 ± 13.5 mL/min/1.73 m^2^ respectively, 63% and 66% of the participants had an eGFR ≥ 60 mL/min/1.73 m^2^ by the MDRD and CKD-EPI equations. The baseline characteristics of the 1,239 participants are shown in Table 1. Participants who sustained an ASVD event over the 10 years of the study had lower baseline values for eGFR by the MDRD and the CKD-EPI equations and higher values for age, body mass index, prevalent diabetes, systolic blood pressure and previous history of ASVD.

**Table 1:** Baseline characteristics of the total cohort and those with and without subsequent atherosclerotic vascular disease (ASVD) hospitalization and mortality

**Baseline Characteristics**	**All participants (n = 1,239)**	**With ASVD (n = 369)**	**Without ASVD (n = 870)**	**P value**
**Age (years)**	75.20 ± 2.71	75.73 ± 2.87	74.97 ± 2.61	< 0.001
**Body mass index (kg/m2)**	27.21 ± 4.68	27.82 ± 5.29	26.95 ± 4.38	0.003
**Systolic blood pressure (mm Hg)**	137.76 ± 18.00	140.73 ± 18.27	136.54 ± 17.76	< 0.001
**Current smoker (yes/no)**	6 (0.0)	3 (0.0)	3 (0.0)	0.371
**Diabetes (yes/no)**	85 (6.9)	38 (10.3)	47 (5.4)	0.002
**Prevalent ASVD (yes/no)**	150 (12.1)	90 (24.4)	60 (6.9)	< 0.001
**MDRD eGFR (mL/min/1.73 m**^**2**^**)**	65.20 ± 14.54	62.42 ± 13.72	66.37 ± 14.73	< 0.001
**CKD-EPI eGFR (mL/min/1.73 m**^**2**^**)**	66.26 ± 13.47	63.54 ± 13.76	67.42 ± 13.19	< 0.001

Results are mean ± SD or number and (%). P values are a comparison of those with and without ASVD hospitalizations at 10 years by univariate ANOVA or Chi squared test where appropriate. ASVD atherosclerotic vascular disease, eGFR estimated glomerular filtration rate, MDRD Modification of Diet in Renal Disease and CKD-EPI Chronic Kidney Disease EPIdemiology.

**Figure 1:**
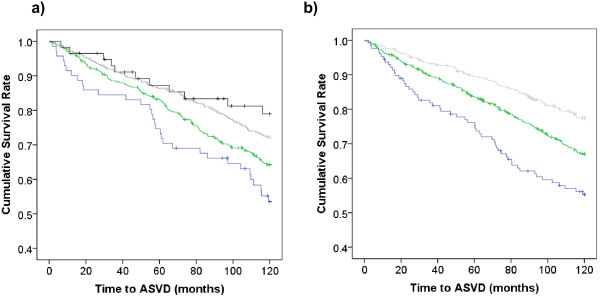
**Kaplan Meier survival curves for ASVD hospitalizations and deaths dichotomized by a) K/DOQI chronic kidney disease categories of eGFR by the MDRD equation; blue line <45 mL/min/1.73 m**^**2**^**(n = 71), green line 45–59 mL/min/1.73 m**^**2**^**(n = 388), grey line 60–89 mL/min/1.73 m**^**2**^**(n = 722) and black line ≥90 mL/min/1.73 m**^**2**^**(n = 58) and b) Framingham predicted risk, blue line ≥30% 10-year risk (n = 128), green line 15-29% 10-year risk (n = 649) and grey line <15% 10-year risk (n = 427).**

The relationship between each individual’s atherosclerotic vascular disease hospitalizations and mortality outcomes and their eGFR, total Framingham score and each component of that score are shown in Table 2. As expected smoking, diabetes and prevalent ASVD showed the largest effects, however because of small numbers current smoking was not significant. eGFR by both methods compared well to the other continuous risk factors systolic blood pressure and BMI. The Kaplan Meir data for ASVD hospitalization and death by categories of (K/DOQI) and Framingham predicted risk are presented in Figure 1. Individuals with baseline calculated eGFR > than 90 mL/min/1.73 m^2^ had a disease free survival of 81% whereas individuals with an eGFR < than 45 mL/min/1.73 m^2^ had a disease free survival of 54%.

**Table 2:** Odds ratios for atherosclerotic vascular disease hospitalizations and mortality by individual variables used in the Framingham risk calculators and eGFR

**Characteristics (n = 1,239)**	**SD**	**Odds Ratio (95% CI)**	**P value**
**Age (years)**	2.71	1.32 (1.17-1.49)	<0.001
**Body mass index (kg/m2)**	4.68	1.20 (1.06-1.35)	0.003
**Current smoker**	yes/no	2.37 (0.48-11.79)	0.292
**Diabetes**	yes/no	2.01 (1.29-3.14)	0.002
**Prevalent ASVD**	yes/no	4.36 (3.05-6.20)	< 0.001
**Systolic blood pressure (mm Hg)**	18.00	1.26 (1.11-1.43)	< 0.001
**MDRD eGFR (mL/min/1.73 m**^**2**^**)**	14.54	1.34 (1.18-1.53)	< 0.001
**CKD-EPI eGFR (mL/min/1.73 m**^**2**^**)**	13.47	1.34 (1.18-1.52)	< 0.001
**Framingham risk score 10-year risk (%)**	8.58	1.45 (1.28-1.65)	< 0.001

Results are unadjusted OR (mean 95% CI) by baseline standard deviation for continuous variables or yes/no for categorical variables. ASVD indicates atherosclerotic vascular disease, eGFR estimated glomerular filtration rate, MDRD Modification of Diet in Renal Disease equation and CKD-EPI Chronic Kidney Disease EPIdemiology equation.

**Figure 2:**
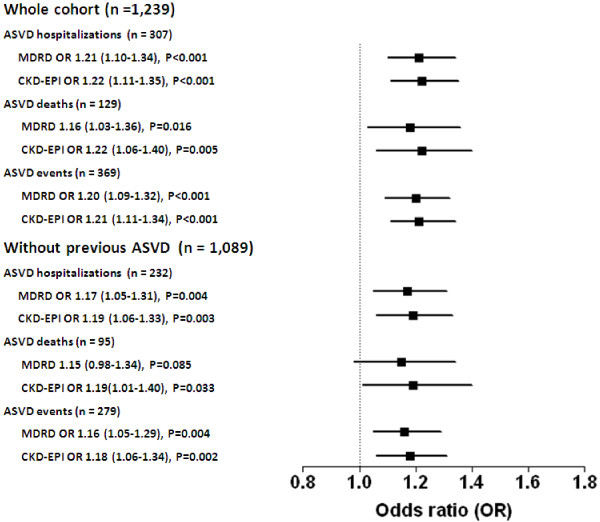
Framingham risk score-adjusted odds ratio and 95% confidence interval per 10 ml/min/1.73 m² decrease in eGFR in the whole cohort and those free of ASVD at baseline. ASVD atherosclerotic vascular disease, eGFR estimated glomerular filtration rate, MDRD Modification.

Next the Framingham risk score and eGFR calculated by the two methods were examined for independent predictive ability for ASVD risk by inclusion of both variables in the same model to predict ASVD outcomes (Table 3). In each of the comparisons both Framingham risk scores and eGFR calculations per SD were significantly associated with ASVD hospitalization or deaths and the combined hospitalizations and deaths when adjusted for the other value. To put these findings in a clinical context the effect of eGFR on ASVD hospitalization and deaths was calculated using 10 mL/min/1.73 m^2^ decreases in eGFR as the unit of change. Analysis of 1,089 participants without ASVD at baseline showed that while the association between eGFR and ASVD hospitalizations and deaths remained significant it was reduced (Figure 2).

**Table 3:** Comparison of eGFR and Framingham risk score in predicting ASVD outcomes in a model including both terms

	**eGFR**	**Framingham**
**ASVD hospitalizations (n = 307)**		
** MDRD**	1.33 (1.15-1.53)	1.48 (1.29-1.69)
** CKD-EPI**	1.31 (1.15-1.50)	1.47 (1.28-1.68)
**ASVD deaths (n = 129)**		
** MDRD**	1.29 (1.05-1.57)	1.37 (1.14-1.64)
** CKD-EPI**	1.32 (1.09-1.59)	1.36 (1.13-1.63)
**ASVD events (n = 369)**		
** MDRD**	1.31 (1.14-1.50)	1.43 (1.26-1.63)
** CKD-EPI**	1.30 (1.15-1.48)	1.43 (1.25-1.62)

The results are odds ratio and 95% confidence interval per SD decrease in eGFR and per SD increase in Framingham risk score includes age, body mass index, smoking, systolic blood pressure and diabetes. ASVD atherosclerotic vascular disease, eGFR estimated glomerular filtration rate, MDRD Modification of Diet in Renal Disease equation, CKD-EPI Chronic Kidney Disease EPIdemiology equation.

The Net Reclassification Improvement (NRI) comparing the 10-year Framingham general cardiovascular risk factors and the MDRD eGFR calculation is shown in Table 4. Using the CKD-EPI eGFR calculation the NRI was 5.1% P = 0.040 and the IDI was 0.015 ± 0.003, P < 0.001.

**Table 4:** Reclassification among people who do or do not experience an ASVD hospitalization or death during 10-year of follow

	**Framingham predicted risk with eGFR by MDRD equation**					
**Framingham predicted risk without eGFR**	**< 15%**	**15-30%**	**≥ 30%**	**Reclassified higher risk**	**Reclassified lower risk**	**Correctly reclassified**
**Participants who experienced ASVD hospitalization or death (n = 352)**						
**< 15%**	0	3	0			
**15-30%**	6	124	23	26 (7.4%)	23 (6.5%)	3 (0.9%)
**≥ 30%**	0	17	179			
**Participants who did not experience ASVD hospitalization or death (n = 852)**						
**< 15%**	14	9	0			
**15-30%**	34	439	50	59 (6.9%)	102 (12.0%)	43 (5.0%)
**≥ 30%**	3	65	238			

Net reclassification improvement (NRI) = 5.9% P = 0.018, IDI = 0.010 ± 0.003, P < 0.001. Framingham risk factors included age, body mass index, smoking, systolic blood pressure and diabetes. ASVD indicates atherosclerotic vascular disease; eGFR estimated glomerular filtration rate and MDRD Modification of Diet in Renal Disease equation.

## Discussion

These data show a relationship between reduction in eGFR by both the MDRD and CKD-EPI equations and increased long-term ASVD-related hospitalization and mortality in elderly women. This association was independent of the method of calculation of eGFR and analytical approach using either per SD reduction or per 10 mL/min/1.73 m^2^ reductions in eGFR. Although slightly diminished by the inclusion of the Framingham risk models the relationship was not abolished, supporting the concept that renal dysfunction may have its deleterious effect via pathways independent of other traditional risk factors associated with increased ASVD risk [1].

Compared with an elderly woman with an eGFR of 90 ml/min/1.73 m², an elderly women with eGFR of 60 ml/min/1.73 m² (currently the reporting level for concern) would be at a 60 - 63% increased risk of long-term ASVD-associated hospitalization or death independent of Framingham risk factors. These findings support and extend previous studies showing increased risk in younger patients with an eGFR 60 – 90 ml/min/1.73 m² [10] . In contrast a recent literature review by Chang and Kramer found eGFR did not significantly improve the C-index in AUC analysis in addition to the Framingham equation [24]. These studies however did not use the newer net reclassification improvement (NRI) or integrated discrimination improvement (IDI) metrics which are designed to evaluate improved predictive ability and are considered to be more powerful than the C-index for comparing predictive models [25]. If addition of variables to a base model improves assignation of individuals to higher or lower probability of having an event the NRI and the IDI value can measure the degree of improvement. The NRI uses clinically relevant cut points while the IDI metrics uses risk as a continuous variable. The inclusion of eGFR calculated by either equation added to the Framingham risk factors improved the NRI by between 5.1 to 5.9%. Direct comparisons to other markers of cardiovascular disease are difficult due to the differences in study population demographics, event rates and duration of study however the overall net reclassification improvement is similar to the 5.3% NRI observed where C reactive protein and parental history of myocardial infarct was added to traditional cardiovascular risk factors for cardiovascular disease risk prediction [22]. The addition of eGFR to the Framingham risk scores also improved the IDI by 0.010 - 0.015. These results are comparable to those reported in a recent study which found that adding eGFR to other known risk factors improved the IDI by 0.011 [26].

The strengths of this study relate to the complete person-based linkage of the 1,239 participants for all atherosclerotic vascular hospitalizations and mortality using previously validated hospital admission and mortality data from the Western Australian Data Linkage System that has been used in over 250 publications [27] as well as the availability of detailed adverse event data. Limitations of the study are that compared to other studies the sample size is small and is restricted to elderly women. Second the use of serum creatinine, rather than serum cystatin C, which has been shown by some [28-31], but not others [32], to be a stronger predictor of all-cause and cardiovascular mortality particularly in older participants. Others have argued that the strong association of cystatin C with all cause and cardiovascular mortality may be due to its association with factors other than GFR such as measures of body size, diabetes and inflammation [33]. Finally data on the albumin creatinine ratio in these patients was not available a measurement that may add significant predictive data [34].

## Conclusion

In this population impaired renal function is an independent risk for atherosclerotic vascular disease in addition to Framingham risk factors possibly related to age-related structural changes in older kidneys resulting in a reduction in functioning glomeruli.

## Abbreviations

ASVD, Atherosclerotic vascular disease; CKD, Chronic kidney Disease; CKD-EPI, Chronic Kidney Disease EPIdemiology; eGFR, Estimated glomerular filtration rate; GFR, Glomerular filtration rate; OR, Odds Ratio; K/DOQI, Kidney disease outcomes quality initiative; MDRD, Modification of Diet in Renal Disease Study.

## Competing interests

The authors have nothing to declare.

## Author contribution

Obtaining funding: JRL, EL, RLP, KZ, WL, conception and design: JL, RLP, WL, data analysis and interpretation - JL, SSD, KZ, RLP, WL and drafting of the manuscript and critical revision: JRL, SSD, EL, RLP, KZ, PLT and WL. All authors’ read and approved the final manuscript.

## Relationship with Industry

None

## Pre-publication history

The pre-publication history for this paper can be accessed here:

http://www.biomedcentral.com/1471-2369/13/58/prepub
